# Resolutions of the Mad River Valley Dental Association

**Published:** 1865-10

**Authors:** 


### RESOLUTIONS OF MAD RIVER VALLEY DENTAL ASSOCIATION.

Whereas, it being made known to the Mad River Valley Dental Association, that our worthy brother and co-laborer, Dr. George Watt, is seriously ill, on motion by Dr. A. A. Blount, it was

*Resolved*, That we deeply deplore our brother's illness, and greatly regret, in consequence thereof, that he cannot be present with us to cheer and aid us, by his large experience and wise counsels, in the discussions of the present sessions of our 3d Quarterly Meeting of this Association.

*Resolved*, That we also extend to himself and family, our profound sympathy, and trust that if it be in accordance with the will of our Heavenly Father, he may be speedily restored to health and his wonted usefulness to our noble profession.

Done in the city of Xenia, Ohio, in session at Dr. G. L. Paine's residence, September 5th, 1865.

GEO. L. PAINE, *President.*

J. H. Paine, *Cor. Secretary.*

---

Mr Editor. *Sir*: — It is but proper and just that I express my appreciation and gratitude to my brethren of the Mad River Valley Dental Association, for the interest and sympathy manifested by them for me in my sickness and suffering. For this as well as for other exhibitions of favor, they will ever be held in grateful remembrance. G. WATT.

